# The ratio of intratumour to stromal infiltrating lymphocytes better predicts prognosis in breast cancer

**DOI:** 10.1002/ctm2.1265

**Published:** 2023-05-22

**Authors:** Baoyi Zhang, Xiang Wang, Chao Cheng

**Affiliations:** ^1^ Department of Chemical and Biomolecular Engineering Rice University Houston Texas USA; ^2^ Quantitative and Computational Biology Program Graduate School of Biomedical Sciences Baylor College of Medicine Houston Texas USA; ^3^ Department of Medicine Baylor College of Medicine Houston Texas USA; ^4^ Institute for Clinical and Translational Research Baylor College of Medicine Houston Texas USA; ^5^ Dan L Duncan Comprehensive Cancer Center Baylor College of Medicine Houston Texas USA

Dear Editor,

Tumour‐infiltrating lymphocytes (TILs) play important roles in the regulation of tumour growth and progression.^1^ It has been established that the presence of TILs was associated with better prognosis and treatment outcomes in different cancer types including breast cancer.^2^, ^3^, ^4^ TILs can be detected within the tumour as intratumour infiltrating lymphocytes (iTILs) and in the adjacent stromal regions as stromal TILs (sTILs). Most of the previous prognostic studies did not distinguish iTILs and sTILs or only focused on iTILs.^5^, ^6^ The prognostic value of sTILs has not been carefully investigated. In this study, we re‐analysed two haematoxylin and eosin (H&E) breast cancer datasets from the FinHER (GSE47994) trial^7^ and TCGA (The Cancer Genome Atlas), respectively. Our results indicate that the differential lymphocytic infiltration between the tumour and adjacent stromal regions (iTIL/sTIL ratio) serves as a better prognostic biomarker compared to iTIL or sTIL alone.

First, we analysed the FinHER dataset (GSE47994), which provided iTIL and sTIL levels determined from H&E‐stained tumour slides as well as major clinical factors for 335 breast cancer patients.^7^ Univariable Cox regression analysis indicated that iTIL but not sTIL was associated with distant recurrence‐free survival (DRFS) (Figure 1A, HR = .83, *p* = .004). Importantly, the iTIL/sTIL ratio achieved the most significant association with DRFS (HR = .77, *p* = 4e − 5). The advantage of iTIL/sTIL ratio over iTIL was more obvious after adjusting for age, tumour size, ER (oestrogen receptor) and HER2 (human epidermal growth factor receptor 2) status with multivariable Cox regression models (Figure 1A, HR = .76 with *p* = 2e − 5 vs. HR = .80 with *p* = .001). Moreover, when all three lymphocyte factors were considered simultaneously in a multivariable model, the iTIL/sTIL ratio was identified as the only prognostic factor (Figure 1B, HR = .80, *p* = .04). Prognostic analyses based on the overall survival (OS) of patients resulted in similar results (Figure S1A).

**FIGURE 1:**
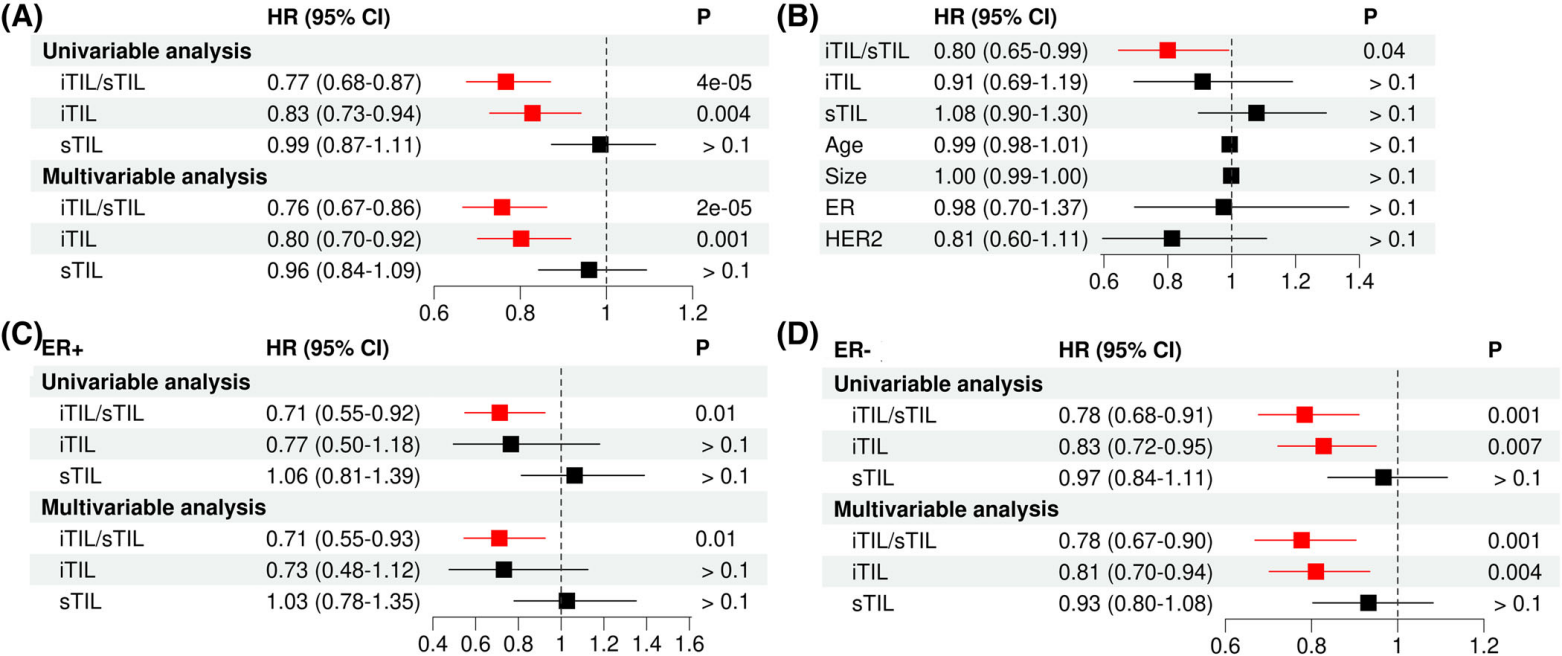
The intratumour infiltrating lymphocytes/stromal tumour‐infiltrating lymphocyte (iTIL/sTIL) ratio is more associated with distant recurrence free survival than iTIL and sTIL in the FinHER dataset. Forest plots demonstrate the hazard ratio (HR) and *p*‐value associated with each factor from different models: (A) univariable and multivariable Cox regression models for iTIL/sTIL, iTIL and sTIL, separately; (B) multivariable Cox regression model, including iTIL/sTIL, iTIL and sTIL, in the same model; (C) the same as (A) but for ER+ patient only and (D) the same as (A) but for ER− patient only. In (A), (C) and (D), age, tumour size, oestrogen receptor (ER) and HER2 status were used as confounding variables in the multivariable analysis. Red indicates a significance level of *p* < .05.

Previous studies have shown that ER status was a critical prognostic factor and correlated with TIL levels in breast cancer.^8^, ^9^ We thus performed stratified analyses based on ER status. In the ER+ subset with 93 patients, both univariable and multivariable analyses indicated that iTIL and sTIL are not prognostic, but the iTIL/sTIL ratio was significantly associated with DRFS (Figure 1C). A similar result was also observed in the ER− subset with 228 patients (Figure 1D). Additionally, we performed stratified analyses based on HER2 status. In the HER2+ subset (*n* = 192), the ratio exhibited a more significant association with DRFS than iTIL according to the results from both univariable (HR = .71 with *p* = 3e − 4 vs. HR = .78, *p* = .01) and multivariable analyses (HR = .71 with *p* = 3e − 4 vs. HR = .76, *p* = .005) (Figure S1B). In the HER2− subset (*n* = 129), similar results were observed (Figure S1C). Taken together, our results indicated that the iTIL/sTIL ratio provided a more significant value for prognostic stratification in breast cancer.

To validate our findings, we analysed the H&E‐stained images and clinical variables that were generated from the TCGA breast cancer (BRCA) data. We applied a deep learning model to separate tumour and adjacent stromal regions in each H&E image. By referring to the study by Abousamra et al.,^10^ we calculated the average lymphocyte densities in the segmentized regions to obtain iTIL and sTIL levels for each patient (see the Supporting Information section). After removing H&E images with low qualities, we obtained the iTIL and sTIL levels for a total of 820 patients. We performed multivariable Cox regression analyses to examine their association with the OS of patients. Again, our results indicate that the iTIL/sTIL ratio (HR = .71, *p* = .01) outperformed iTIL (HR = .80, *p* = .04) and sTIL (*p* > .1) levels in predicting OS after adjusting for age, tumour stage and ER status (Figure 2A). In the model including iTIL, sTIL and their ratio as competitive predictors, the iTIL/sTIL ratio was found to be the only factor that remained significant after adjusting for the above‐described clinical factors (Figure 2B, HR = .54, *p* = .006).

**FIGURE 2:**
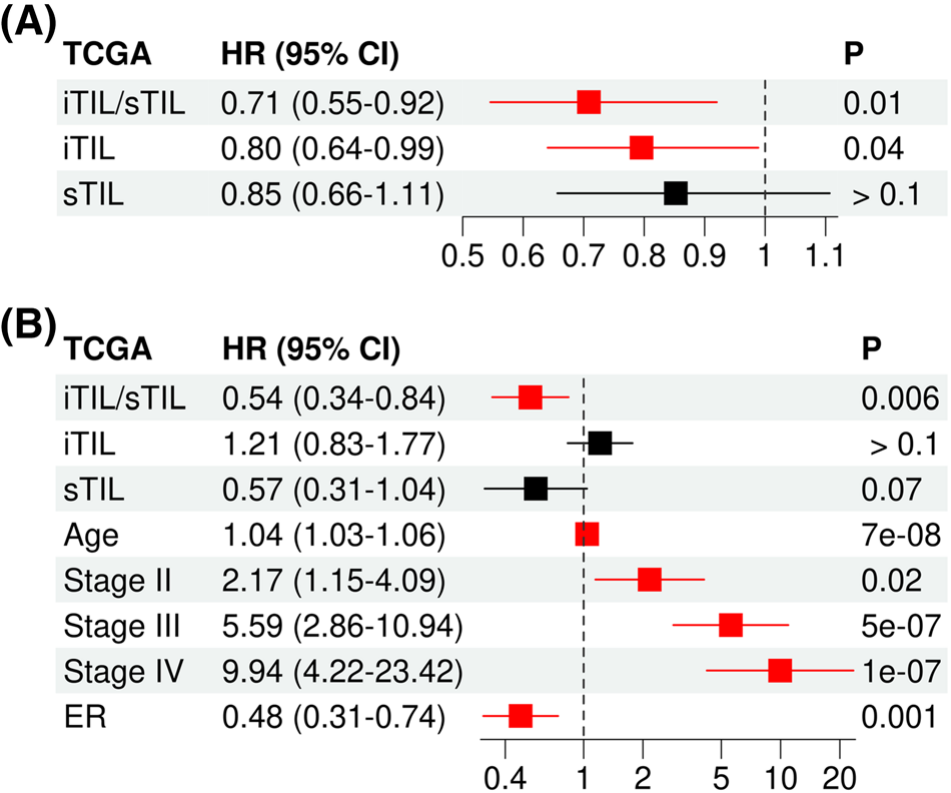
The intratumour infiltrating lymphocytes/stromal tumour‐infiltrating lymphocyte (iTIL/sTIL) ratio is more associated with overall survival than iTIL and sTIL in the TCGA (The Cancer Genome Atlas) breast cancer dataset: (A) results from multivariable Cox regression models that investigate iTIL/sTIL, iTIL and sTIL separately, while adjusting for age, stage and oestrogen receptor (ER) status; (B) results from multivariable analysis, including iTIL/sTIL, iTIL, and sTIL, as competing factors. Red indicates a significance level of *p* < .05. HR, hazard ratio.

In conclusion, our study indicates the iTIL/sTIL ratio is more associated with prognosis compared iTILs and sTILs (Figure 3). The iTIL and sTIL levels are highly correlated (e.g. with a correlation coefficient of .73 in the GSE47994 dataset), and both indicate the immune hotness of the tumour microenvironment. However, in both datasets, the iTIL but not the sTIL level was significantly associated with prognosis, suggesting that lymphocytes infiltrating into the tumour posed a stronger prognostic impact than their stromal counterparts. Although the sTIL level reflects the baseline immune infiltration of a tumour, the iTIL/sTIL ratio might indicate the ability of lymphocytes to further approach tumour cells, especially those inside the tumour. In other words, how exclusive the tumour cells are to surrounding TILs. As such, iTIL/sTIL ratio is more prognostic, which serves as a better biomarker than TILs.

**FIGURE 3:**
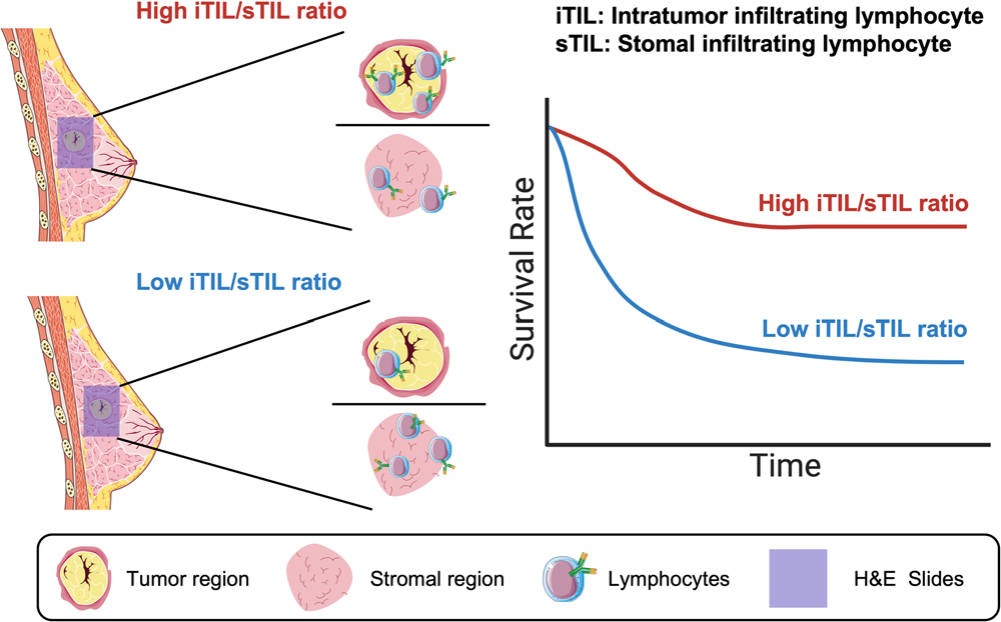
Intratumour infiltrating lymphocytes/stromal tumour‐infiltrating lymphocyte (iTIL/sTIL) ratio reflects the infiltration level of lymphocytes from stromal to tumour region, thereby being more significantly associated with patient prognosis. H&E, haematoxylin and eosin. *Source*: Parts of the figure were drawn by using pictures from Servier Medical Art. Servier Medical Art by Servier is licensed under a Creative Commons Attribution 3.0 Unported License (https://creativecommons.org/licenses/by/3.0/).

## CONFLICT OF INTEREST STATEMENT

The authors declare no potential conflicts of interest.

## FUNDING INFORMATION

Cancer Prevention Research Institute of Texas (CPRIT), Grant No.: RR180061 and National Cancer Institute of the National Institutes of Health, Grant No.: 1R01CA269764

## Supporting information

Supporting Information

Supporting Information
