# Supplementary figures and images for: The ratio of intratumour to stromal infiltrating lymphocytes better predicts prognosis in breast cancer
Source: Clin Transl Med. 2023 May 22;13(5):e1265. doi: 10.1002/ctm2.1265 (PMC10203537; doi:10.1002/ctm2.1265)

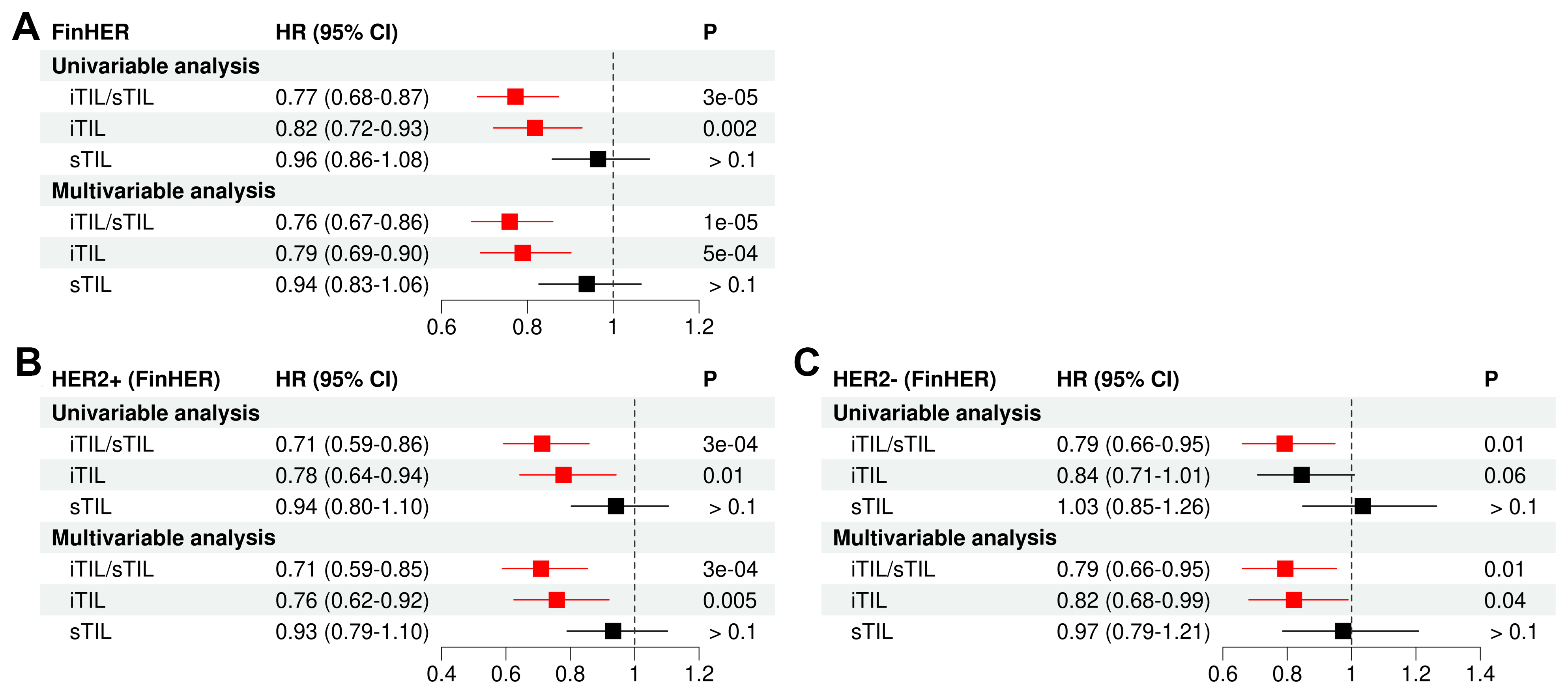

Supplement: Supplementary file 1 — Supporting Information [file CTM2-13-e1265-s002.tif]
