# Supplementary material for: The ratio of intratumour to stromal infiltrating lymphocytes better predicts prognosis in breast cancer
Source: Clin Transl Med. 2023 May 22;13(5):e1265. doi: 10.1002/ctm2.1265 (PMC10203537; doi:10.1002/ctm2.1265)
Supplement: Supplementary file 2 — Supporting Information [file CTM2-13-e1265-s001.docx]

**Supplementary methods**

**Patient cohorts**

The FinHER trial [1] dataset was downloaded from Gene Expression Omnibus (GEO) database with the accession ID GSE47994, which provides curated intratumor infiltrating lymphocyte (iTIL), and stromal infiltrating lymphocyte (sTIL) levels for 335 breast cancer patients. The iTIL and sTIL values were calculated from full-face hematoxylin and eosin-stained (H&E) tumor slide sections by pathologists [1]. Major clinical factors such as age, tumor size, ER and HER2 status were also included.

As for The Cancer Genome Atlas Breast Cancer (TCGA-BRCA) cohort, we downloaded 1133 H&E diagnostic slide images from the Genomic Data Commons Portal (<https://portal.gdc.cancer.gov/>). We calculated the iTIL and sTIL for each patient by referring to a previous publication [2].

**Image preprocessing**

The TCGA-BRCA data provides scanned H&E whole slide images. Only slides with a magnification greater than 20x were included in this study. We resized all TCGA slides into 20x magnification and tessellated them into square tiles of 512x512 pixels (corresponding to 250x250 μm) using OpenSlide [3]. We considered pixels with RGB values over 220 as backgrounds and discarded tiles with over 50% backgrounds.

**Tumor and stromal region detection in TCGA**

We first developed a deep learning model to detect tumor and stromal regions in all slides from TCGA data. Specifically, we used the BreAst Cancer Histology images (BACH) [4] data to develop a tumor versus stroma classification model; and then applied the developed model to classify TCGA tiles into either tumor or stroma. The BACH data contains 400 images of 1536x2048 pixels (20x magnification) from four classes: normal, benign, *in situ* carcinoma and invasive carcinoma (100 images per class). To match the input size of TCGA tiles, we tessellated each of the BACH images into 12 tiles of 512x512 pixels.

We randomly divided the BACH tiles into train, validation, and test sets at 7:1:2 on the patient level. To control overfitting, we applied image augmentations including random horizontal, vertical flip, and random translation. We merged normal and benign classes as stroma, and *in situ* carcinoma and invasive carcinoma class as tumor; and trained a model with ResNet18 backbone to classify the two classes using binary cross entropy loss. We started the learning rate at 1e-4, which decays to one-tenth of the previous value after 3 epochs of non-decreasing loss in the validation set. Finally, we achieved an AUC of 0.97 and accuracies for tumor and stromal classes of 0.89 and 0.93 in the test set, respectively.

Having shown the high performance of the developed model, we then applied it to classify all TCGA-BRCA tiles in each slide as either tumor or stroma tile.

**Calculation of iTIL and sTIL**

A previous study provides regional TIL density in TCGA-BRCA data as TIL maps [2]. The TIL maps provide probabilities of small regions (50x50 μm) across each slide predicted to be infiltrating-lymphocytes positive from a deep learning model [2]. By matching our predicted tumor and stroma regions with their TIL maps, we calculated mean TIL density, represented by the mean predicted probabilities, in tumor and stromal regions for every patient as their iTIL and sTIL. 170 patients have less than 50 tumor or adjacent stromal tiles. These patients as well as 10 male patients were excluded from the downstream analysis to ensure the data quality. Finally, we calculated iTIL and sTIL levels for 820 patients in TCGA-BRCA data.

**Survival analysis**

Univariable Cox regression was used to estimate the prognostic value of iTIL, sTIL and iTIL/sTIL. Multivariable Cox regression was used to examine the adjusted prognostic value of iTIL, sTIL and iTIL/sTIL after consideration of established clinical factors including age, tumor size, ER and HER2 status.

**Supplementary figures**


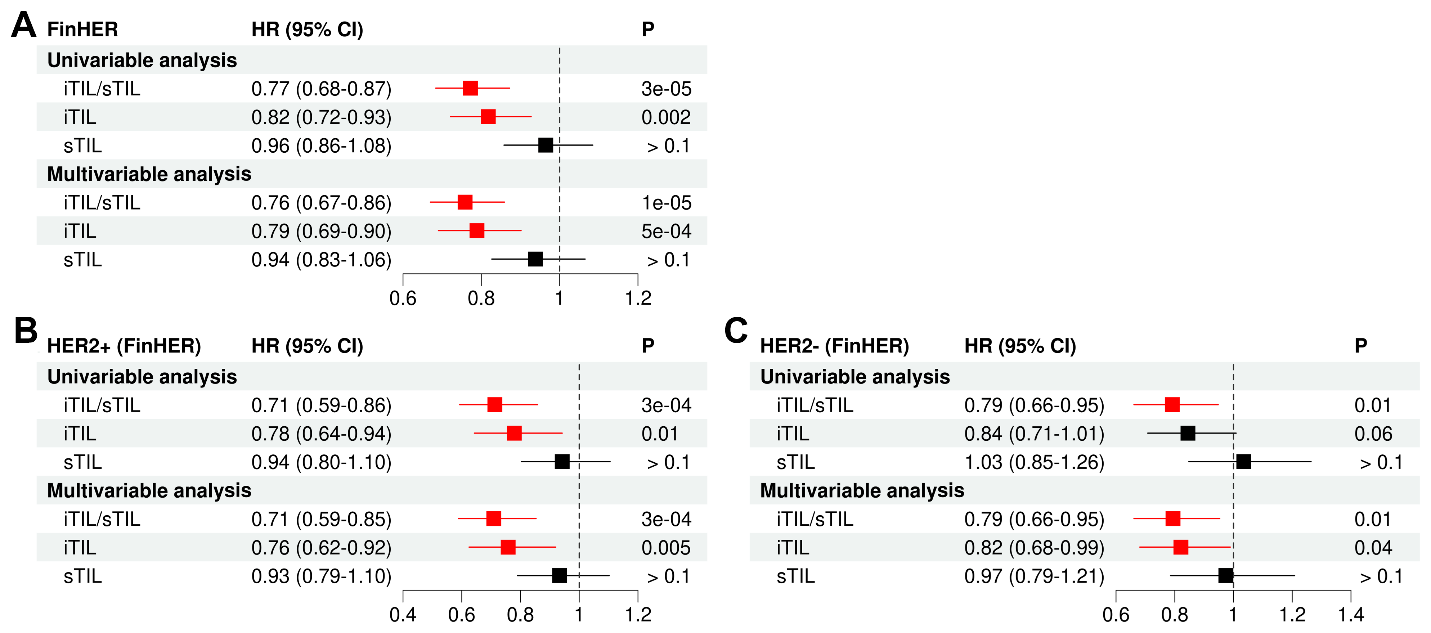


**Figure S1: Association of the iTIL/sTIL ratio with prognosis in the FinHER dataset. (A)** The iTIL/sTIL ratio is more predictive of patient overall survival. **(B)** Association of iTIL/sTIL, iTIL, and sTIL with distant recurrence free survival in HER+ patients. **(C)** the same as B but in HER- patients. In the multivariable analysis, age, tumor size, ER and HER2 status were used as confounding variables. Red color indicates a significance level of p < 0.05.

**Reference**

[1] S. Loi *et al.*, “Tumor infiltrating lymphocytes are prognostic in triple negative breast cancer and predictive for trastuzumab benefit in early breast cancer: results from the FinHER trial,” *Ann Oncol*, vol. 25, no. 8, pp. 1544–1550, Aug. 2014, doi: 10.1093/annonc/mdu112.

[2] S. Abousamra *et al.*, “Deep Learning-Based Mapping of Tumor Infiltrating Lymphocytes in Whole Slide Images of 23 Types of Cancer,” *Front Oncol*, vol. 11, p. 806603, 2021, doi: 10.3389/fonc.2021.806603.

[3] A. Goode, B. Gilbert, J. Harkes, D. Jukic, and M. Satyanarayanan, “OpenSlide: A vendor-neutral software foundation for digital pathology,” *J Pathol Inform*, vol. 4, p. 27, 2013, doi: 10.4103/2153-3539.119005.

[4] G. Aresta *et al.*, “BACH: Grand challenge on breast cancer histology images,” *Med Image Anal*, vol. 56, pp. 122–139, Aug. 2019, doi: 10.1016/j.media.2019.05.010.
